# Percutaneous endoscopic interlaminar discectomy for L5-S1 calcified lumbar disc herniation: A retrospective study

**DOI:** 10.3389/fsurg.2022.998231

**Published:** 2022-09-23

**Authors:** Yuanpei Cheng, Qianru Zhang, Yongbo Li, Xipeng Chen, Han Wu

**Affiliations:** ^1^ Department of Orthopedics, China-Japan Union Hospital of Jilin University, Changchun, China; ^2^ Department of Cardiology, Shanghai Ninth People's Hospital, Shanghai Jiaotong University School of Medicine, Shanghai, China

**Keywords:** calcified lumbar disc herniation, lumbar disc herniation, percutaneous endoscopic interlaminar discectomy, percutaneous endoscopic transforaminal discectomy, clinical efficacy

## Abstract

**Background:**

Calcified lumbar disc herniation (CLDH) is considered to be a special type of lumbar disc herniation (LDH). Percutaneous endoscopic interlaminar discectomy (PEID), with safety and efficacy, has been proved to be a minimally invasive surgery for LDH. However, there are few studies on PEID in the treatment of CLDH at the L5-S1 level. This study aimed to analyze the clinical efficacy of PEID for L5-S1 CLDH.

**Methods:**

From August 2016 to April 2020, we retrospectively analyzed 28 consecutive patients (17 males and 11 females) with L5-S1 CLDH treated with PEID at our institution. All the patients were monitored for more than 1 year postoperatively. The demographic characteristics, surgical results, and clinical outcomes estimated by the visual analog scale (VAS) for leg pain, the Oswestry disability index (ODI), and the modified MacNab criteria were collected.

**Results:**

All patients successfully underwent PEID. The mean operative time and intraoperative blood loss were 65.36 ± 5.26 min and 13.21 ± 4.35 ml, respectively. The VAS for leg pain and ODI scores improved remarkably from 7.54 ± 0.96 to 1.50 ± 0.51 (*P* < 0.05) and from 69.29 ± 9.91 to 17.43 ± 3.69 (*P* < 0.05) a year after operation, respectively. According to the modified MacNab criteria of the last follow-up, the excellent and good rates are 92.86%. Two of the patients had complications, one had nerve root injury and the other had postoperative dysesthesia.

**Conclusions:**

PEID achieved good clinical outcomes in the treatment of L5-S1 CLDH, and it was a safe and effective minimally invasive surgery for L5-S1 CLDH.

## Introduction

Calcified lumbar disc herniation (CLDH) is considered a special type of lumbar disc herniation (LDH). Baron was the first to report a case of intervertebral disc calcification in 1924 (1). However, the etiology of intervertebral disc calcification is still unclear (2, 3). The main symptoms of CLDH include severe leg pain, lower limb numbness, and sometimes lower limb weakness. More seriously, calcified discs may cause dural tear and nerve root injury (4). Patients with CLDH should be treated *via* surgical operation after the failure of conservative treatments such as drugs, bed rest, and physical therapy (5).

Traditional open surgery, with the complete removal of a calcified disc, the sufficient decompression of spinal canal, and the loosening of nerve root, is often used to treat CLDH in clinic (6). Regardless of the satisfactory clinical outcomes, traditional open surgery has some drawbacks, including large tissue damage, long operation time, considerable blood loss, slow postoperative recovery, and even muscle denervation and atrophy (7). Some studies (8, 9) indicated that traditional open surgery may cause long-term complications such as spinal stenosis and spondylolisthesis.

Percutaneous endoscopic interlaminar discectomy (PEID), with a short incision, less trauma, little intraoperative blood loss, and fast postoperative recovery, is a minimally invasive surgical procedure. PEID, with safety and effectiveness, has been proved to have a comparable clinical efficacy to the traditional open surgery in the treatment of soft LDH (10, 11). However, it is difficult to treat CLDH with PEID because the calcified disc is hard and tightly adheres to nerve root and the dural sac. With the development of spinal endoscopic instruments such as ultrasonic osteotomes, PEID is gradually used in the treatment of CLDH. However, there are few reports on PEID in the treatment of CLDH at the L5-S1 level (12–14). The purpose of the research is to discuss the clinical efficacy of PEID for L5-S1 CLDH and provide clinical guidance for spinal surgeons.

## Methods

### General information

The study received the support of the Ethics Committee of our institution and informed consent of all patients, and was also in accordance with the Helsinki declaration. From August 2016 to April 2020, 28 consecutive patients with L5-S1 CLDH underwent PEID at our institution. Informed consents of all patients were obtained before they were included in the study. The inclusion criteria were as follows: (1) lower extremity pain and numbness; (2) LDH with calcification demonstrated by computed tomography (CT) and magnetic resonance imaging (MRI); (3) limited to the L5-S1 segment; (4) failure of conservative treatments for more than 3 months; (5) the follow-up time more than 12 months. The exclusion criteria were as follows: (1) LDH without calcification; (2) multisegmental lesions; (3) non-L5-S1 segment; (4) combination with spinal stenosis, instability, tuberculosis, infection, or tumor; (5) patients not fit for operation due to some medical comorbidities. A percutaneous interlaminar endoscopic spine system (Joimax, Karlsruhe, Germany), ultrasonic osteotome (SMTP, China) and a tip-flexible bipolar radiofrequency system (Elliquence LLC, USA) were used during the operation (15, 16).

### Surgical procedure

**Figure 1:**
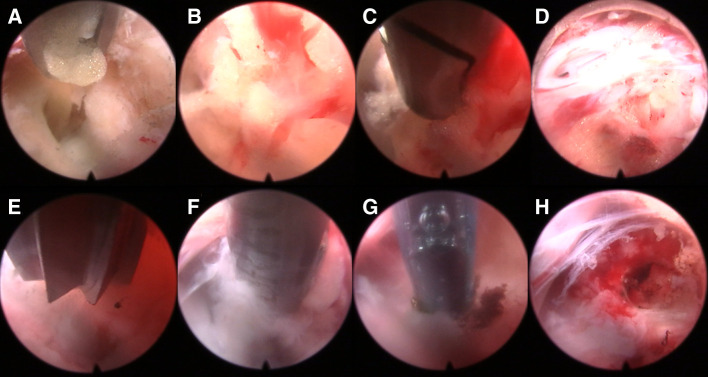
Endoscopic images of PEID (**A–H**). (**A**) The ligamentum flavum was cut by scissors. (**B**) Spinal epidural lipomatosis was found. (**C**) Spinal epidural lipomatosis was removed. (**D**) The nerve root and dural sac were exposed. (**E**) An ultrasonic osteotome was used to cut the calcified and herniated disc. (**F**) The calcified and herniated disc was removed by a nucleus pulposus forceps. (**G**) Bipolar radiofrequency was used to hemostasis. (**H**) The uncompressed nerve roots and dural sac were found.

**Figure 2:**
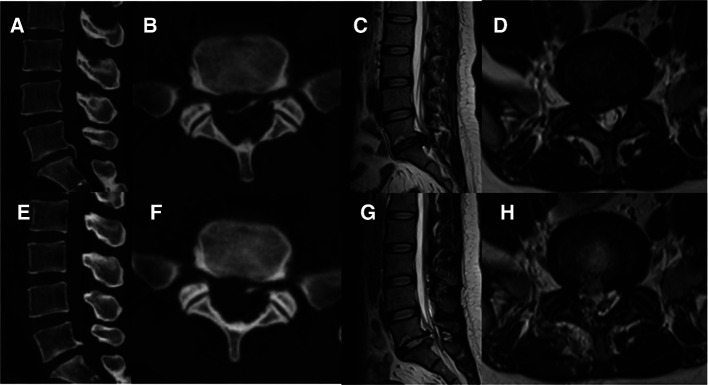
Pre- and post-operative CT and MRI (**A-H**). (**A-D**) Preoperative CT and MRI revealed lumbar disc herniation combined with calcification. (**E-H**) Postoperative CT and MRI revealed that the calcified intervertebral disc was removed and the compressed nerve root had been relieved by PEID.

**Figure 3:**
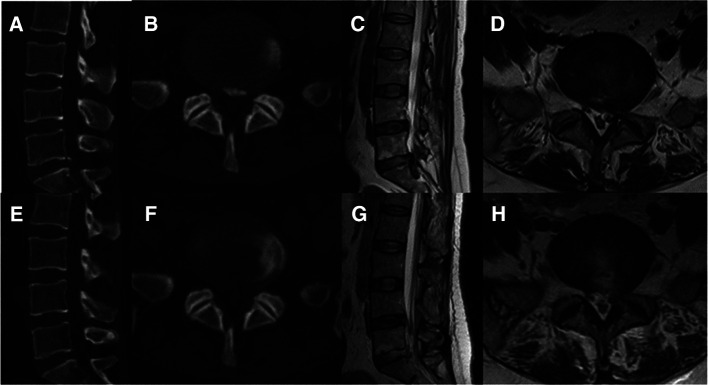
Pre- and post-operative CT and MRI (**A-H**). (**A-D**) Preoperative CT and MRI revealed lumbar disc herniation combined with calcification. (**E-H**) Postoperative CT and MRI revealed that the calcified intervertebral disc was removed and the compressed nerve root had been relieved by PEID.

The patient was placed on the operating table in the prone position, and the procedure was performed under general anesthesia. The operative segment was identified under fluoroscopic guidance. The entry point on the body surface was determined at 1.0 cm from the posterior midline. The 18-gauge puncture needle was inserted at the L5-S1 level under fluoroscopic guidance, and the target position was the lateral edge of the interlaminar space. The guidewire, blunt dilator, and working cannula were introduced in turn. After connecting and checking the operating system, the operation was conducted under endoscopic visualization. A 0.7 cm incision was made. Under the endoscope, the ligamentum flavum was cut by the scissor (Figure 1A). After removing the fat from the epidural space, the nerve root and dural sac were exposed (Figures 1B–D). The position of dural sac, compressed nerve root, and herniated disc was defined before the removal of the calcified intervertebral disc. Under the endoscope, the calcified disc was carefully removed with the ultrasonic osteotome (SMTP, China), and the herniated disc fragment was removed using the endoscopic forceps (Figures 1E,F). Bleeding sites were carefully explored and given sufficient hemostasis (Figure 1G). Finally, no obvious compression of the nerve root and dural sac was observed under endoscopic visualization (Figure 1H). The working cannula was removed. All patients underwent CT scans and MRI before and after operation (Figures 2, 3).

### Measures

Demographic characteristics, including age, sex, body mass index (BMI), and follow-up time, were collected. Surgical outcomes, such as operative time, intraoperative blood loss, intraoperative fluoroscopy times, postoperative hospital stay, and complications, were recorded. Clinical outcomes, including the visual analog scale (VAS) (17) for leg pain, the Oswestry disability index (ODI) (18), and the modified MacNab criteria (19), were investigated.

### Statistical assessments

Clinical outcomes were statistically analyzed by the IBM SPSS Version 24 software (IBM Corporation, Armonk, New York, USA). The results were presented as mean and standard deviation (SD) values calculated from the data. Statistical analysis was carried out *via* repeated measures analysis of variance. *P* < 0.05 indicated a statistically significant difference.

## Results

### Demographic characteristics and surgical outcomes

All patients successfully underwent PEID by the same experienced surgeon. We monitored all 28 patients (17 male and 11 female) for at least 12 months (range, 12–24 months). The demographic characteristics (age, sex, BMI, and follow-up time) and surgical outcomes (the operative time, intraoperative fluoroscopy times, postoperative hospital stay, and intraoperative blood loss) of all patients are shown in Table 1. The mean operative time, intraoperative fluoroscopy times, postoperative hospital stay, and intraoperative blood loss were 65.36 ± 5.26 min, 2.96 ± 0.88 times, 2.64 ± 1.16 days, and 13.21 ± 4.35 ml, respectively.

**Table 1:** Demographic characteristics and surgical outcomes of all patients.

Variables	Value
Patients (number)	28
Age (years)	38.61 ± 8.79
Sex (male/female)	17/11
BMI (kg/m^2^)	25.03 ± 3.27
Follow-up (months)	15.21 ± 2.64
Operative time (minutes)	65.36 ± 5.26
Fluoroscopy times (n)	2.96 ± 0.88
Intraoperative blood loss (ml)	13.21 ± 4.35
Postoperative hospital stay (days)	2.64 ± 1.16
Complications	2 (7.14%)

Values are mean ± SD, number, or as otherwise indicated.

BMI, body mass index.

### Clinical outcomes

**Figure 4:**
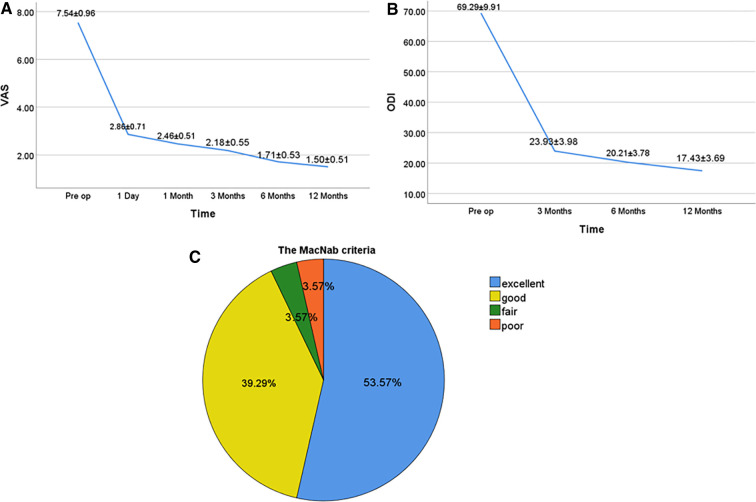
Clinical outcomes at different follow-up time points for pre- and postoperative PEID (**A–C**). (**A**) VAS scores for pre- and postoperative PEID. (**B**) ODI scores for pre- and postoperative PEID. (**C**) The modified MacNab criteria of all patients. *P* < 0.05. VAS, visual analog scale; ODI, Oswestry disability index; Pre op, preoperation.

The VAS scores of the all patients were 2.86 ± 0.71, 2.46 ± 0.51, 2.18 ± 0.55, 1.71 ± 0.53, and 1.50 ± 0.51 at 1 day postoperatively, 1, 3, 6, and 12 months postoperatively, respectively, which were significantly lower than those preoperatively (7.54 ± 0.96), with statistically significant differences (*P* < 0.05 for all; Figure 4A). The ODI scores of all patients were 23.93 ± 3.98, 20.21 ± 3.78, and 17.43 ± 3.69 at 3, 6, and 12 months postoperatively, which were significantly lower than those preoperatively (69.29 ± 9.91), with statistically significant differences (*P* < 0.05 for all; Figure 4B). Based on the modified MacNab criteria at the last follow-up, 15 cases were excellent, 11 cases were good, 1 case was fair, and 1 case was poor. The excellent and good rates were 92.86% (Figure 4C).

### Complications

In the study, one patient had nerve root injury and one had postoperative dysesthesia. The two patients recovered after conservative treatments. During the follow-up period, no complications such as dural tear, cerebrospinal fluid leakage, epidural hematoma, or infection were observed.

## Discussion

CLDH, with a low incidence rate, is a relatively rare type of LDH (20, 21). Although the pathogenesis is still uncertain, a study demonstrated that intervertebral disc calcification may be caused by some factors such as inflammation, trauma, and blood supply disruption (2, 3). However, the symptoms in adults are difficult to relieve or even worsen after conservative treatments such as nonsteroidal anti-inflammatory drugs, bed rest, physiotherapy, and epidural steroid injection (5, 22).

Some surgical techniques, with good clinical results, are applied to the treatment of CLDH (6, 23–26). Traditional open surgery, with complete removal of calcified disc, is the most commonly used surgical procedure (6). However, some complications of traditional open surgery, such as dural tear, cerebrospinal fluid leakage, incision infection, long-term chronic low back pain, and spinal instability, still need to be widely concerned (8, 9, 27, 28). Some studies reported that percutaneous endoscopic transforaminal discectomy (PETD) was used to treat CLDH and achieved good clinical outcomes (14, 29). Yu et al. (16) reported that 25 CLDH patients were treated by PETD with ultrasonic osteotomes. Unfortunately, they found that seven patients had postoperative dysesthesia and one patient had recurrence of herniation. Shin et al. (30) showed that PETD achieved a good clinical result in the treatment of CLDH. However, there were two serious complications of dural tear. There was a high iliac crest at the L5-S1 level, which increased both the difficulty and the incidence of complications of PETD. Moreover, PEID had a long and difficult learning curve, which limited the use of the procedure and increased the risk of surgical complications (31).

In this study, PEID combined with ultrasonic osteotome was performed to treat CLDH at the L5-S1 level. Compared with those before surgery, the VAS and ODI scores of all patients at any follow-up time point after surgery were significantly improved (Figures 3A,B). The symptoms of all patients disappeared or were significantly relieved, and no serious complications occurred during the follow-up period. We concluded that PEID achieved good clinical outcomes in the treatment of CLDH at the L5-S1 level with safety and effectiveness. The incision length was only 0.7 cm, the intraoperative blood loss was only 13.21 ml, and the hospital stay was only 2.64 days, which proved that PEID was a minimally invasive procedure (Table 1). There is a large laminar space at the L5-S1 level, which is the natural advantage of PEID (32). Under endoscopic visualization, PEID was performed to remove the calcified disc easily and release the compressed nerve root sufficiently, which minimized the damage of the nerve root and dural sac (12). The calcified disc adhered to the nerve root and dural sac, which increased the risk of nerve root injury and dural sac tear when the calcified disc was removed (33). Calcified intervertebral disc was safely and effectively removed with ultrasonic osteotome during operation in our study, which reduced the risk of nerve root injury and dural tear. Ultrasonic osteotome has some characteristics of tissue selectivity, antirolling, hemostasis, and easy operation (34–37). According to our experience, the important points were as follows. First, the location and size of the calcified disc were accurately evaluated by preoperative lumbar CT and MRI examinations. Second, a safe working area was first made by removing epidural adipose tissue and soft intervertebral disc. Third, a clear surgical view was ensured by timely and adequate hemostasis during operation. Forth, violent separation, exposure, and removal of the calcified disc were avoided, as these operations tended to cause nerve root injury and dural tear due to the adhesions of the calcified disc to the nerve root and dural sac. Fifth, once the compressed nerve root and dural sac were completely released, excessive removal of the calcified disc was not recommended, as it increased the risk of complications such as nerve root injury and dural tear.

There were some previous studies on PEID in the treatment of CLDH (12–14). Dabo et al. (12) reported that 30 patients with CLDH were treated by PEID. They concluded that nerve root traction due to rotation of the trephine may cause postoperative dysesthesia when the calcified disc was removed. Kim et al. (13) reported that PEID with calcification floating technique was performed to treat some patients with CLDH and achieved good clinical outcomes. Nevertheless, the method of rotating the working channel to separate the calcified disc was perhaps inefficient. It also caused nerve root injury and dural tear when the calcified disc was tightly adhered to the nerve root and dural sac. Chen et al. (14) reported that 13 patients with CLDH underwent PEID with the Peak method. Among the patients treated with PEID, only one patient had a dural tear and cerebrospinal fluid leakage due to the adhesion. However, laminotomy and facetectomy were conducted to expose the peak of calcification, which increased both the difficulty and the trauma of the procedure. In our study, however, PEID combined with ultrasonic osteotome was performed to solve the problems of the trephine rotation and the adhesions of the calcified disc to the nerve root and dural sac, reduce the risk of the procedure greatly, and improve the clinical effects significantly.

There were some limitations in this retrospective study. First, this study had a small sample size and lacked a control group. Second, the short follow-up period did not evaluate the long-term efficacy of our surgical method. Prospective randomized controlled trials with large sample size, multicenter, and long-term follow-up are still needed to better evaluate the clinical efficacy of this procedure in the future.

## Conclusion

PEID was a safe and effective minimally invasive procedure and achieved good clinical outcomes in the treatment of L5-S1 CLDH. We believed that PEID might be used as an alternative to traditional open surgery for CLDH at the L5-S1 level.

## Data Availability

The raw data supporting the conclusions of this article will be made available by the authors, without undue reservation.
